# miR-150 Suppresses the Proliferation and Tumorigenicity of Leukemia Stem Cells by Targeting the Nanog Signaling Pathway

**DOI:** 10.3389/fphar.2016.00439

**Published:** 2016-11-18

**Authors:** Dan-dan Xu, Peng-jun Zhou, Ying Wang, Yi Zhang, Rong Zhang, Li Zhang, Su-hong Chen, Wu-yu Fu, Bi-bo Ruan, Hai-peng Xu, Chao-zhi Hu, Lu Tian, Jin-hong Qin, Sheng Wang, Xiao Wang, Qiu-ying Liu, Zhe Ren, Xue-kui Gu, Yao-he Li, Zhong Liu, Yi-fei Wang

**Affiliations:** ^1^College of Life Science and Technology, Jinan UniversityGuangzhou, China; ^2^College of Biology Technolgy, Guangdong Food and Drug Vocational CollegeGuangzhou, China; ^3^Faculty of Environmental and Biological Engineering, Guangdong University of Petrochemical TechnologyMaoming, China; ^4^Section of Otolaryngology, Department of Surgery, Yale School of Medicine, New HavenCT, USA; ^5^State Key Laboratory of Oncology in South China and Collaborative Innovation Center for Cancer Medicine, Sun Yat-sen University Cancer CenterGuangzhou, China; ^6^The First Affiliated Hospital, Guangzhou Hospital of Traditional Chinese MedicineGuangzhou, China

**Keywords:** miR-150, proliferation, tumorigenicity, Nanog, leukemia stem cells

## Abstract

Proliferation, a key feature of cancer cells, accounts for the majority of cancer-related diseases resulting in mortality. MicroRNAs (miRNAs) plays important post-transcriptional modulation roles by acting on multiple signaling pathways, but the underlying mechanism in proliferation and tumorigenicity is unclear. Here, we identified the role of miR-150 in proliferation and tumorigenicity in leukemia stem cells (LSCs; CD34+CD38- cells). miR-150 expression was significantly down-regulated in LSCs from leukemia cell lines and clinical samples. Functional assays demonstrated that increased miR-150 expression inhibited proliferation and clonal and clonogenic growth, enhanced chemosensitivity, and attenuated tumorigenic activity of LSCs *in vitro*. Transplantation animal studies revealed that miR-150 overexpression progressively abrogates tumor growth. Immunohistochemistry assays demonstrated that miR-150 overexpression enhanced caspase-3 level and reduced Ki-67 level. Moreover, luciferase reporter assays indicated Nanog is a direct and functional target of miR-150. Nanog silencing using small interfering RNA recapitulated anti-proliferation and tumorigenicity inhibition effects. Furthermore, miR-150 directly down-regulated the expression of other cancer stem cell factors including Notch2 and CTNNB1. These results provide insights into the specific biological behavior of miR-150 in regulating LSC proliferation and tumorigenicity. Targeting this miR-150/Nanog axis would be a helpful therapeutic strategy to treat acute myeloid leukemia.

## Introduction

Acute myeloid leukemia is a malignant haematologic disease characterized by an aberrant accumulation of immature myeloid cells. Although progress has been made, several important issues remain, including resistance and disease relapse. Evidence demonstrates that AML is organized as a hierarchy of several distinct leukaemic blasts with different self-renewal and differentiation potentials (Bonnet and Dick, 1997). Studies from clinical data and experimental systems have shown that AML originates from a rare population of LSCs (CD34+CD38- cells) or leukemia-initiating cells (LICs) which are capable of self-renewal, proliferation, and differentiation into malignant blasts (Lapidot et al., 1994; Bonnet and Dick, 1997; Roboz and Guzman, 2009). LSCs share some antigenic features with normal haematopoetic stem cells (HSCs), such as CD34+, CD38-, CD71-, and HLA-DR-, but can be phenotypically distinguished from HSCs by disparate markers (Lapidot et al., 1994; Blair et al., 1997, 1998; Bonnet and Dick, 1997; Jordan et al., 2000). These cells are responsible for therapeutic resistance and are drivers of disease progression and relapse (Jordan et al., 2006; Ishikawa et al., 2007). Recent studies have demonstrated that several solid tumors are heterogeneous cell populations and are maintained by cancer stem cells (CSCs) with higher tumorigenic potential (Ginestier et al., 2007; Chiou et al., 2010; Choi et al., 2012; Du et al., 2013; Boumahdi et al., 2014). Consequently, it is reasonable that targeting CSCs is essential for cancer disease treatment.

miRNAs are conserved non-coding RNAs of 18–25 nucleotides in length that suppress gene expression at the post-transcriptional level by blocking mRNA translation or degrading target mRNAs through its binding to the 3′-untranslated regions (3′-UTRs) of target genes (Bartel, 2004). Mounting evidence has shown that the abnormal expression of miRNAs or mutations correlates with various human cancers and have been identified as unique signatures associated with diagnosis, staging, prognosis, and response to treatment (Esquela-Kerscher and Slack, 2006; Wang et al., 2015). In addition, miRNAs function as tumor suppressors and oncogenes (Esquela-Kerscher and Slack, 2006). Recently, emerging evidence suggests that miR-150 functions as a major regulator in determining the fate of haematopoietic stem/progenitor cells in both lymphoid and myeloid lineages (He Y. et al., 2014). For examples, downregulation of miR-150 is observed in CML, AML, and lymphoma, whereas its upregulation has been reported in MDS and CLL (Fulci et al., 2007; Agirre et al., 2008; Hussein et al., 2010; Zhao et al., 2010; Fayyad-Kazan et al., 2013). The critical tumor suppressor role of miR-150 has been demonstrated in the pathogenesis of AML, particularly MLL gene-rearranged AML (Jiang et al., 2012). miR-150 is a critical tumor suppressor and gatekeeper in leukaemogenesis and its repression is required for the development of *MLL*-rearranged AML (Jiang et al., 2012). Furthermore, functional studies illustrated that miR-150 is also an essential tumor suppressor in lymphoma (Watanabe et al., 2011). MYB, FLT3, EGR2 are important target genes of miR-150 in AML and lymphoma (Watanabe et al., 2011; Jiang et al., 2012; Bousquet et al., 2013). Although miR-150 downregulation expression has been observed in hematopoietic disease, including CML, AML, CLL, and MDS, its definitive pathological role remains to be elucidated (He Y. et al., 2014). In addition, the mechanism underlying the role of miR-150 in regulating LSC proliferation and tumorigenicity has not been fully elucidated.

Nanog, a homeodomain protein, is required for the pluripotency of embryonic stem cells (ESCs) and it, along with Oct4 and Sox2, forms a core ESC network (Mitsui et al., 2003; Boyer et al., 2005). In addition, functional studies provide evidence that Nanog plays a vital role in malignant disease, correlating with cell proliferation and various malevolent properties such as clonogenic growth, tumorigenicity, invasiveness, and therapeutic resistance (Noh et al., 2012; Shan et al., 2012; Jeter et al., 2015). Clinical studies revealed that Nanog is overexpressed in a variety of cancers (Zbinden et al., 2010; Choi et al., 2012; Noh et al., 2012; Shan et al., 2012; Jeter et al., 2015). Moreover, Nanog was found to be highly expressed in oesophageal cancer tissues and was positively correlated with histological grade and lymphatic metastases (Yang et al., 2012). According to Yang’s studies, Nanog could promote tumor cell proliferation, invasion, and resistance (Yang et al., 2012). In addition, Eberle et al. (2010) provided evidence that Nanog is expressed in AML cells (Eberle et al., 2010). These studies are important and form the base of this study.

In this study, we investigated the biological function and role, and the underlying molecular mechanism of miR-150 in LSC proliferation and tumorigenicity. We identified significant downregulation of miR-150. Furthermore, enhanced expression of miR-150 potently inhibited proliferation and promoted apoptosis *in vitro* and *in vivo*. In addition, Nanog has been identified as a functional and direct target of miR-150. We further demonstrated that si-Nanog, using small interfering RNA (siRNA), recapitulated the effect of miR-150 on proliferation inhibition. Finally, we confirmed that miR-150 downregulated other key factors, including Notch2 and CTNNB1.

## Materials and Methods

### Cell Culture

KG-1a and MOLM13 cell lines were obtained from the Laboratory Animal Center of Sun Yat-sen University. The cells were cultured in RPMI 1640 medium supplemented with 10% FBS and 1% penicillin-streptomycin at 37°C in 5% CO2. KG-1a-LSCs (CD34+CD38-) and MOLM13-LSCs (CD34+CD38-) were isolated using magnetic microbeads (Miltenyi Biotec, Germany) and the purity of LSCs was 93.58% as described in our previous study (Zhang et al., 2015). Human leukemia samples were collected in the First Affiliated Hospital of Guangdong Traditional Medicine University (FAHGTMU). All the materials were obtained with written informed consent, and the procedures were approved by the FAHGTMU and the Ethical Committee of Jinan University.

### qPCR Analysis and miRNA Detection

The total mRNAs were extracted using Trizol (Invitrogen, USA) and were reverse-transcribed using a Bio-Rad system. qPCR was performed on a Bio-Rad system using Taqman for mRNAs and miRNAs. Expression of miR-150 was analyzed using the SsoFast EvaGreen Supermix miRNA detection Kit (Bio-Rad, CA). U6 expression was used as an internal control. miR-150 expression in each sample was calculated by normalizing with U6 and the relative expression was calculated using 2^-ΔΔCt^ values. mRNA expression level in Nanog, Notch2, Hsp90B1 and CTNNB1 were analyzed by the primers described in **Supplementary Table S1**. β-actin was used as an internal control. These stemness genes expression level were calculated using 2^-ΔΔCt^ values. All the experiments were done in triplicate. A *t*-test was used to evaluate the differences of miR-150 and those stemness genes expression levels among two groups. A *P* value of < 0.05 was considered significant. SPSS 19.0 was employed to calculate the difference.

### Western Blot Analysis

Cells were lysed and the protein concentration was determined using BCA assay (Beyotime Biotechnology, China). The protein were subjected to polyacrylamide gel electrophoresis. Then, the proteins were transferred to a polyvinylidene fluoride (PVDF) membrane (Millipore, USA). The membrane was blocked with blocking buffer [0.1% Tween-20 in 5% skimmed milk (Gibco, USA)] for 1.5 h. The membrane was incubated with a primary antibody [Nanog, Cell Signaling Technology (CST), #3580; β-catenin, CST, #9582; Hsp90B1, CST, #20292; Notch2, abcam, ab#8927] at 4°C overnight. Next, the membrane was washed with 0.1% Tween-20 Tris-buffered saline. Then, the membrane was incubated with horseradish peroxidase-conjugated secondary antibodies for 1.5 h at room temperature. The bound antibodies were detected using a chemiluminescence detection kit ECL (Millipore, USA). β-actin was used as an internal control.

### Soft Agar and Sphere Formation Assay

For the soft agar assay, 1 × 10^3^ LSCs were mixed with 0.3% low melting agar in IMDM medium (Stem Cell Technologies) supplemented with 10% FBS and plated on a 0.6% low melting agar-coated 6-well plate. The plates were incubated at 37°C in a humidified incubator for 15 days. Every well was stained with 0.2 ml 0.05% crystal violet for 0.5 h at 37°C. The numbers of positive colonies (>8 cells/colony) were counted. The experiments were performed at least three times.

### Flow Cytometry and Annexin V-APC/7-AAD Staining

For the apoptosis assay, cells were transfected with miR-150, miR-NC, si-Nanog, or si-NC for 48 h. They were then harvested and 5 μl of binding reagent and 5 μl of Annexin V-APC (KeyGen BioTech, China) were added. After 30 min, cells were washed three times with PBS and stained with 5 μl of 7-AAD (KeyGen BioTech, China) for 15 min at room temperature according to the manufacturer’s instructions. The detailed steps were guided by the manufacturer’s instructions. The experiments were repeated three times. All data were analyzed and calculated using FlowJo software.

### Luciferase Reporter Assay

The dual-luciferase reporter assay (Promega) was employed to evaluate the interaction between miR-150 and the 3′-UTR of Nanog. The sequences of the Nanog 3′-UTR and the mutant Nanog 3′-UTR were cloned into the luciferase reporter pGL4.11. In addition, miR-150 was cloned into an miRNA expression lentivial vector (Genepharma, China). LSCs (1 × 10^5^ cells/well) were cultured in 24-well plates, transfected with Nanog 3′-UTR and the mutant 3′-UTR of Nanog and either miR-150 or a negative control (NC) using Lipofectamine 2000 (Invitrogen, USA) according to the manufacturer’s protocol. Luciferase activity was measured 48 h after transfection using the Dual Luciferase Reporter Assay System (Beyotime Biotechnology, Haimen, China) and was normalized to Renilla luciferase activity. Wild-type and mutant sequence of Nanog 3′-UTR are seen in **Supplementary Table S1**.

### miRNA Mimic and Inhibition and shRNA Lentiviral Vector Construction

To knockdown the expression of Nanog in LSCs, the Nanog-specific si-Nanog1 and si-Nanog2 was synthesized (Genepharma, China) according to the study of Kyung Hee Noh (Noh et al., 2012). The LSCs were transfected with si-Nanog1 and siNanog2 and siRNA-NC (Genepharma, China) mixed with Lipofectamine 2000 (Invitrogen, USA) according to the manufacturer’s instructions. After transfection for 72 h, total protein and RNAs were prepared from the cells and were subjected to western blot analysis and qPCR, respectively. The sequence of si-Nanog1 and si-Nanog2 and siRNA-NC are seen in **Supplementary Table S1**. In addition, miR-150 lentiviral vector was constructed by Genepharma. The transfections were conducted according to the manufacturer’s protocol. miR-150 sponge and Nanog vector (pcDNA3.1-Nanog) was purchased from Genepharma (Shanghai, China).

### Proliferation Analysis

Leukemia stem cell proliferation analysis was conducted with trypan blue (Beyotime, Haimen, China). LSCs were seeded into a 48-well plate containing 100 μl of medium. Subsequently, the 48-well plate was incubated for 4 h at 37°C in 5% CO_2_ in a humidified incubator. The LSCs were counted by trypan blue. For Ki-67 cell proliferation detection, 1.0 × 10^5^ transfected LSCs were used to complete the experiments. The cells were incubated with an antibody (Cell Signaling Technology) against Ki-67 and were washed three times with Tris-buffered containing 0.1% Tween-20. Then, the cells were incubated with FITC-conjugated goat secondary antibody for 0.5 h. The cells were photographed by a fluorescence microscope (Zeiss, Germany). The data are presented as the mean ± standard deviation (SD) from three independently repeated experiments.

### Animal Models and Immunohistochemistry (IHC)

BALB/c and nonobese diabetic/severe combined immunodeficient (NOD/SCID) mice were housed and bred in specific pathogen-free conditions. All procedures involving animals were approved by the experimental animal center of Jinan University and the Ethical Committee of Jinan University. All animal experiments protocol were conducted according to the animal care ethical guidelines of the Review Committee for the Use of Human or Animal Subjects of Jinan University.

For the subcutaneous model, six 5-week-old female BALB/c mice (HFK Bioscience, Beijing, China) were subcutaneously injected in the neck flank with 2.0 × 10^5^ LSCs in which lentiviral vectors containing miR-150 or NC were transfected. The LSCs were mixed with Matrigel (Corning, USA) at the ratio of 1:2. The tumor size was measured every 3 days, and the tumor volume was calculated as *L* × *W*^2^ × 0.5 (mm^3^; *L* indicates length, and *W* indicates width). Four weeks later, the mice were euthanized, the tumors were harvested and the weight of tumors was measured. The tumors were embedded in paraffin. Slides were pre-treated with citrate buffer (pH 6.0). For blocking endogenous peroxidase activity, the slides were treated for 15 min with methanol containing 0.3% H_2_O_2_. After washing in Tris buffer, the slides were incubated with anti-human Caspase-3 primary antibody (goat polyclonal, diluted 1:200; Abcam, USA) and anti-Ki-67 primary antibody (mouse polyclonal, diluted 1:200, Cell Signaling Technology, USA). For immunostaining, a peroxidase-conjugated antibody was used. IHC was done as described previously (Ferretti et al., 2012).

For malignant hematologic tumor model, four 5-week-old female NOD/SCID mice (*n* = 4) were intravenously injected via the tail vein with 2 × 10^5^ LSCs in which miR-150 was overexpressed by lentiviral vector with GFP tag. In the NC group (*n* = 4), mice were intravenously injected via the tail vein with 2 × 10^5^ LSCs transduced with NC lentiviral vectors. *In vivo* imaging experiments was completed (Xtreme, Germany) and mice were euthanized after 60 days, the spleens were collected. Human CD45+ (hCD45+) leukemia cells from mice spleen were evaluated by flow cytometry (FACS Calibur, BD Company). Human leukemia cells were identified as CD45+ cells.

### Statistical Analysis

Statistical analysis was performed using SPSS 19.0. The experiments were repeated at least three times. The results were presented as the mean ± SD. A two-tailed Student’s *t*-test was used for statistical analysis. Two-way ANOVA was used to determine statistical differences for *in vivo* experiments. ^∗^*P* < 0.05 and ^∗∗^*P* < 0.01 were considered statistically significant in all cases.

## Results

### Overexpression of miR-150 Inhibits LSC Proliferation *In vitro*

**FIGURE 1:**
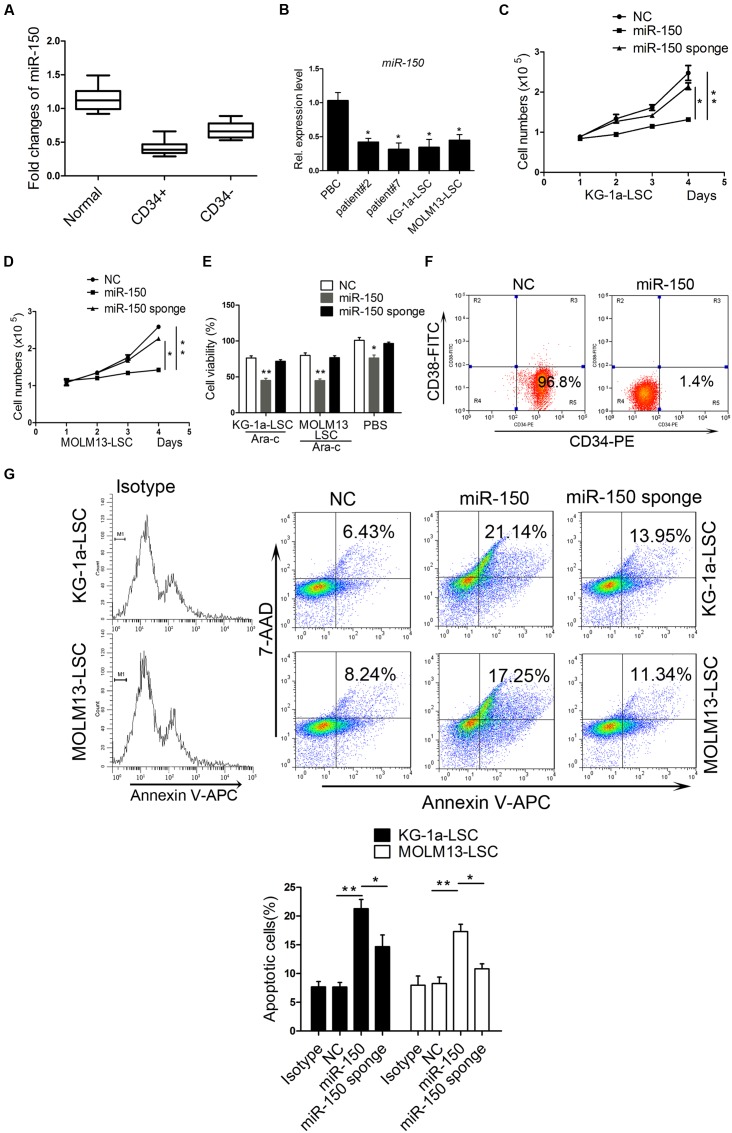
**miR-150 is upregulated in LSCs and plays an essential role in regulating proliferation.** **(A)** qPCR analysis of miR-150 in normal blood and paired CD34+/CD34- cells in clinical samples from AML patients. CD34+ cells were isolated using magnetic microbeads. **(B)** qPCR analysis of miR-150 in another AML patient and LSCs from leukemia cell lines KG-1a and MOLM13. U6 was referenced as a control. **(C,D)** Proliferation analysis of LSCs after miR-150 and NC transfection. 5000 cells were transfected with miR-150 and seeded into 48-well plates. They were cultured at 37°C. The proliferation LSCs was counnted by trypan blue. **(E)** Cell viability analysis of LSCs transfected with miR-150 and NC. LSCs (1.0 × 10^4^ cells/well) were seeded into 96-wells plates and Ara-C was added. Cells were incubated for 48 h. The readings were recorded by a microplate absorbance reader. **(F)** Flow cytometery analysis percentage of KG-1a-LSCs after they were transduced with miR-150 mimic. **(G)** Flow cytometry apoptosis analysis of LSCs transfected with miR-150, miR-150 sponge and NC. ^∗^*P* values of < 0.05 and ^∗∗^*P* values of < 0.01 were considered statistically significant. The experiments were repeated three times independently (mean ±*SD*). NC, negative control.

The miR-150 expression level was reported to be downregulated in leukemia (Fulci et al., 2007; Hussein et al., 2010). To investigate its expression condition and biological function, we collected blood samples from 19 AML patients and 11 healthy subjects and then isolated CD34+/CD34- cells (**Supplementary Table S2**). Consistent with previous studies, on quantification of miR-150 mRNA expression levels using qPCR, we found that the miR-150 level was significantly lower in CD34+ cells than in CD34- cells in patients and normal blood cells (**Figure 1A**). Furthermore, miR-150 expression was downregulated in KG-1a-LSCs (CD34+CD38-) and MOLM13-LSCs (CD34+CD38-) compared with that in normal blood cells (**Figure 1B**). To further confirm its low expression level in leukemia, another two AML clinical samples were collected. Consistently, miR-150 levels were still found to be lower in these two samples (**Figure 1B**). These observations promoted us to investigate its biological function by performing gain-of-function studies. To this end, LSCs isolated form KG-1a and MOLM13 were transfected with miR-150 mimic or NC and cell proliferation was analyzed. Cell transfected with miR-150 (72 h) markedly increased miR-150 levels (**Supplementary Figure S1**). As expected, the proliferation of LSCs transfected with miR-150 was remarkably suppressed compared with that of LSCs transfected with NC (**Figures 1C,D**). On the contrary, miR-150 sponge increased the proliferation effect on LSCs (**Figures 1C,D**). Then, cell viability was evaluated using 5 μM cytarabine (Ara-C), a drug for AML treatment according to our previous studies (Zhang et al., 2015). Cell viability was more inhibited after transfection with miR-150 compared with that with NC after the addition of Ara-C (**Figure 1E**). However, the viability of LSCs transfected miR-150 sponge was increased (**Figure 1E**). These studies demonstrate that miR-150 recovery inhibits LSCs proliferation.

To explore the effect of miR-150 on the proliferation of CD34+CD38- cells, we used CD34-PE and CD38-FITC antibodies to stain the KG-1a-LSCs after transfection of miR-150 or NC and performed flow cytometry analysis. As shown in **Figure 1F**, the percentage of CD34+CD38- cells was decreased significantly in miR-150-transfected cells (1.4%) compared with the NC group (96.8%; **Figure 1F**). To further confirm the inhibiting effects of miR-150 on proliferation, apoptosis experiments were performed. Flow cytometry assays showed that the apoptosis rate increased remarkably in KG-1a-LSCs transfected with miR-150 compared to those transfected with NC (34.71% vs. 8.96%, **Figure 1G**). miR-150 sponge increased the proliferation (**Figure 1G**). Similarly, miR-150 overexpression inhibited MOLM13-LSCs survival (32.63% vs. 9.31%, **Figure 1G**) and miR-150 sponge increased the proliferation (**Figure 1G**). These results suggest that miR-150 suppresses LSCs proliferation.

### miR-150 Overexpression Impairs LSC Clonogenic and Sphere Formation Activities

**FIGURE 2:**
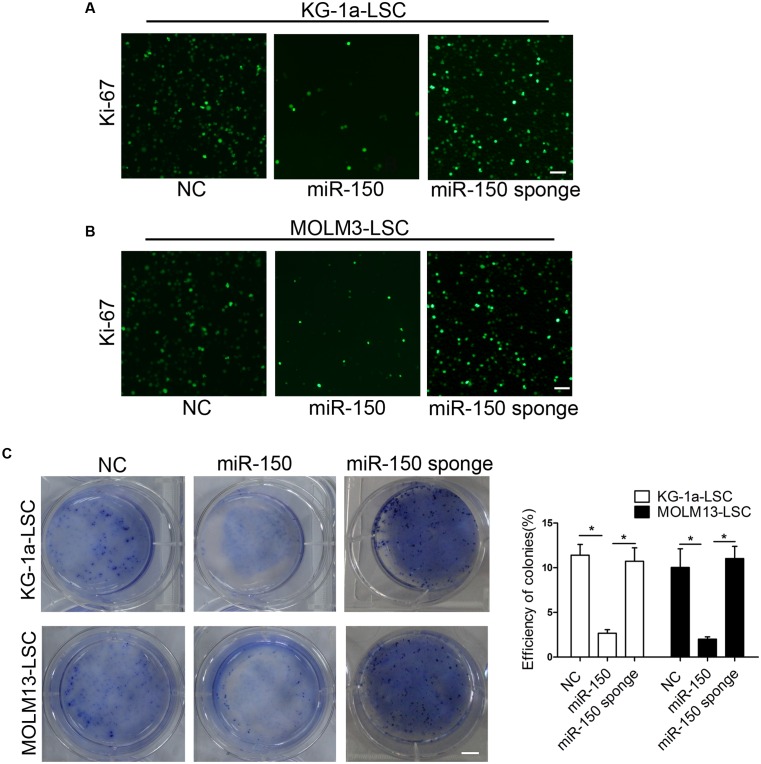
**miR-150 overexpression suppresses LSC clonogenic growth, and sphere-forming capacities.** **(A,B)** Proliferation analysis of LSCs transfected with miR-150 or NC. Transfected LSCs (1.0 × 10^5^ cells) were used to complete the experiments. Cells were incubated with antibody against Ki-67. Cells were washed three times with Tris-buffer containing 0.1% Tween-20. Then, the cells were incubated with goat secondary antibody. Scale bars: 40 μm. **(C)** Soft agar plate experiments were used to test the LSCs proliferation capacities. 1 × 10^3^ LSCs transduced with either miR-150, miR-150 sponge and NC were seeded into a 6-well soft agar plate and cultured for 14 days. The cells were stained with 0.05% crystal violet for 0.5 h at 37°C. Representative micrographs are shown. All data represent the mean ±*SD* from three independent experiments (^∗^*P* < 0.05). In all experiments, data represent the mean ±*SD* from three independent experiments (^∗^*P* < 0.05). Scale bars: 10 mm.

The immunofluorescence assay demonstrated that miR-150 inhibited KG-1a-LSC proliferation according to the expression of Ki-67 (**Figure 2A**). Similarly, the inhibited effect was observed in MOLM13-LSCs (**Figure 2B**). But miR-150 sponge increased Ki-67 expression level (**Figures 2A,B**). The ability to form spheroids in soft agar plate is a property of CSCs. To investigate whether miR-150 regulates clonogenic and sphere formation ability, LSCs were transfected with miR-150 and a soft agar assay was used to assess the regulation of LSCs by miR-150. Our findings demonstrated that miR-150 overexpression greatly decreased, but its inhibition increased, the number of both types LSCs from KG-1a and MOLM13 compared with the NC (**Figure 2C**). In conclusion, these findings revealed that miR-150 overexpression impairs the clonogenic and sphere formation activities.

### miR-150 Overexpression Suppresses Xenograft Tumor Growth *In vivo*

**FIGURE 3:**
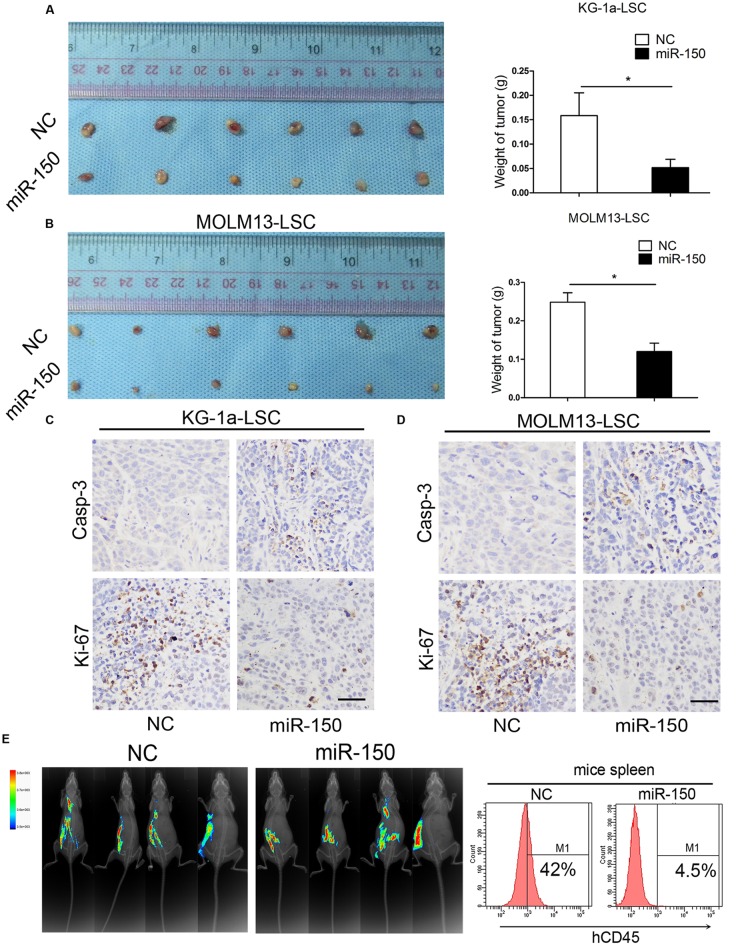
**miR-150 overexpression impairs the tumorigenicity of LSCs.** **(A,B)** Xenograft tumors volume derived from miR-150 and NC lentiviral vectors (Left). LSCs (2.0 × 10^5)^ were transduced with miR-150 or NC. Tumors volume were weighed and photographed after 30 days (Right; mean ± *SD*, ^∗^*P* < 0.05). **(C,D)** Immunohistochemistry analysis of tumor growth. Ki-67 and Casp-3 were detected. Error bars represent the mean ± *SD* from three independent experiments. Scale bars: 40 μm. **(E)** By experimental proliferation and tumorigenicity assay in NOD/SCID mice which were evaluated by fluorescence imaging, flow cytometery analysis demonstrated that miR-150 inhibited LSCs proliferation, whereas NC did not (*n* = 4).

To further help elucidating the suppressed effect of miR-150, we assessed the impact of miR-150 on KG-1a and MOLM13 LSCs *in vivo*. The lentiviral vector encoding miR-150-transduced LSCs from KG-1a were implanted subcutaneously into BALB/c mice, and observations and measurements were recorded 28 days after injection. Strikingly, tumor regeneration was notably inhibited in every case (**Figure 3A**, Left), and tumor growth was severely delayed (**Figure 3A**, right). Consistent with LSC-enriched KG-1a cells, these findings were also observed in MOLM13-LSCs. The findings demonstrated that miR-150 overexpression significantly suppressed tumor growth (**Figure 3B**, left). Moreover, in comparison with the control groups, the weight of the tumors in the group treated with miR-150 were smaller than NC group (**Figure 3B**, right). To further explore the biological role of miR-150, the immunohistochemical staining of Ki-67 and activation of caspase-3 in the tumors was performed. The results revealed that the expression of caspase-3 was increased in the miR-150 overexpression tumor group (**Figures 3C,D**). Meanwhile, decreased Ki-67 expression was observed in miR-150-transduced tumor (**Figures 3C,D**). The *in vivo* role of miR-150 was evaluated by NOD/SCID mice. According to other studies (Guzman et al., 2002; Lumkul et al., 2002), the percenatage of human CD45+ (hCD45+) cells was analyzed by flow cytometry. The percentage of hCD45+ cells in mice spleen was significantly less than the NC group (4.5% vs. 42%; **Figure 3E**). These results implicated that miR-150 inhibited the LSCs proliferation and the tumorigenicity from KG-1a.

### Nanog Is a Functional Target of miR-150

**FIGURE 4:**
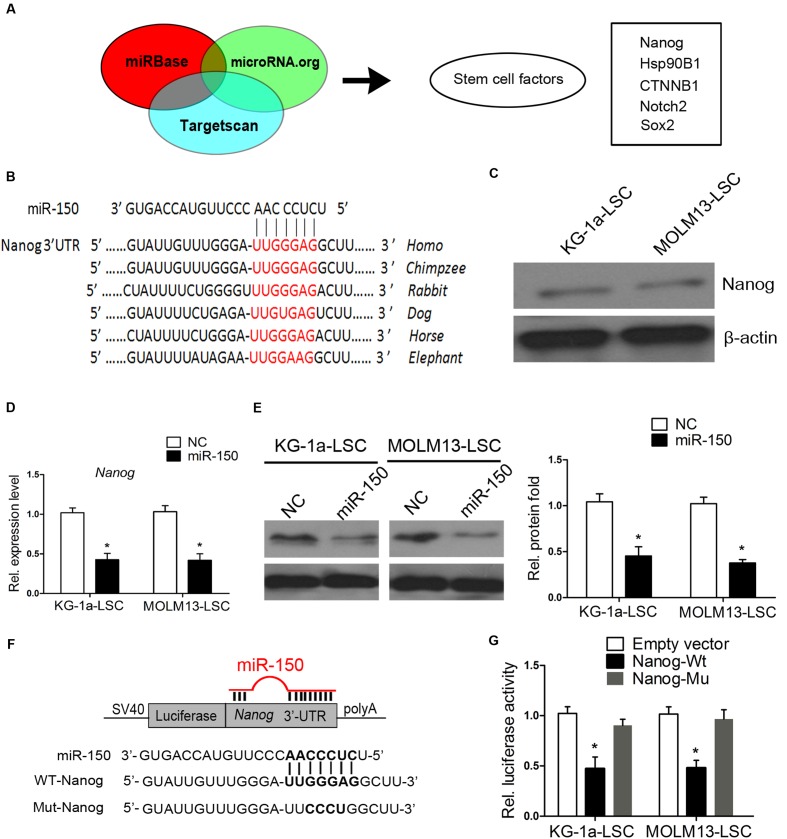
**Nanog is a direct target of miR-150.** **(A)** Diagram analysis of miR-150 target genes predicted by three microRNA research databases. The target genes contain stemness genes. **(B)** Schematic diagram displaying the evolutionarily conservative sites of 3′-UTR targeted by miR-150 among different species. **(C)** Western blot analysis of Nanog in LSCs. β-actin was a loading control. **(D,E)** Nanog mRNA levels and protein expression levels, analyzed by western blot and qPCR 48 h after transfection. β-actin was used as an internal control (^∗^*P* < 0.05). **(F)** Schematic diagram of the Nanog 3′-UTR constructs and the alignment of wild-type (WT) and mutant type (Mut) miR-150 putative target sites in the 3′-UTR of Nanog. **(G)** LSCs were co-transfected with an empty vector (Ctrl), or a wild-type or mutant target site of the Nanog 3′-UTR vector, as well as a miR-150 vector. Luciferase activity was normalized to Renilla activity and presented as relative to miR-NC (^∗^*P* < 0.05). Data for each condition are shown from three independent experiments (mean ±*SD*).

To determine the molecular mechanism by which miR-150 exerts its tumor suppressing properties, we employed 3 algorithms to predict the potential miRNA targets of miR-150, including Targetscan, miRBase, and microRNA.org. These different computational methods identified many candidate genes that were commonly predicted to be possible targets of miR-150. To further narrow the possible downstream effectors of miR-150, Gene Ontology Analysis was carried out. Several stemness genes were found, including Nanog, CTNNB1 coding protein β-catenin, Notch2, and Hsp90B1 (**Figure 4A**). As known, these genes play a critical role in CSCs. We were particularly interested in Nanog because Nanog is critical for ESCs and CSCs (Mitsui et al., 2003; Zbinden et al., 2010; Choi et al., 2012; Shan et al., 2012). In addition, analysis of the 3′-UTR of Nanog showed that it is highly conserved among different species (**Figure 4B**).

We found that Nanog is expressed in KG-1a-LSCs and MOLM13-LSCs (**Figure 4C**). This is consistent with Eberle’s studies (Eberle et al., 2010). To assess whether Nanog is a target of miR-150, we again transfected miR-150 into both of the LSCs and examined Nanog protein and mRNA expression levels. As shown in **Figure 4D**, the mRNA levels of Nanog in the LSCs were sharply decreased compared with those of NC (*P* < 0.05). Consistently, the protein level of Nanog was significantly reduced after transfection with miR-150 (**Figure 4E**). To determine whether Nanog is a direct and functional target of miR-150, we engineered 3′-UTR fragments, in which wild-type and mutant binding sites were inserted into the region downstream of the luciferase reporter gene (**Figure 4F**). Luciferase reporter assays showed that miR-150 transfection caused a notable decrease in relative luciferase activity in LSCs when the Nanog plasmid containing a wild-type 3′-UTR was present (**Figure 4G**). However, the luciferase activity in the 3′-UTR of the mutant binding site did not decrease significantly (**Figure 4G**). These results provided evidence suggested that Nanog is a target of miR-150.

### Downregulation of Nanog Inhibits LSCs Proliferation

**FIGURE 5:**
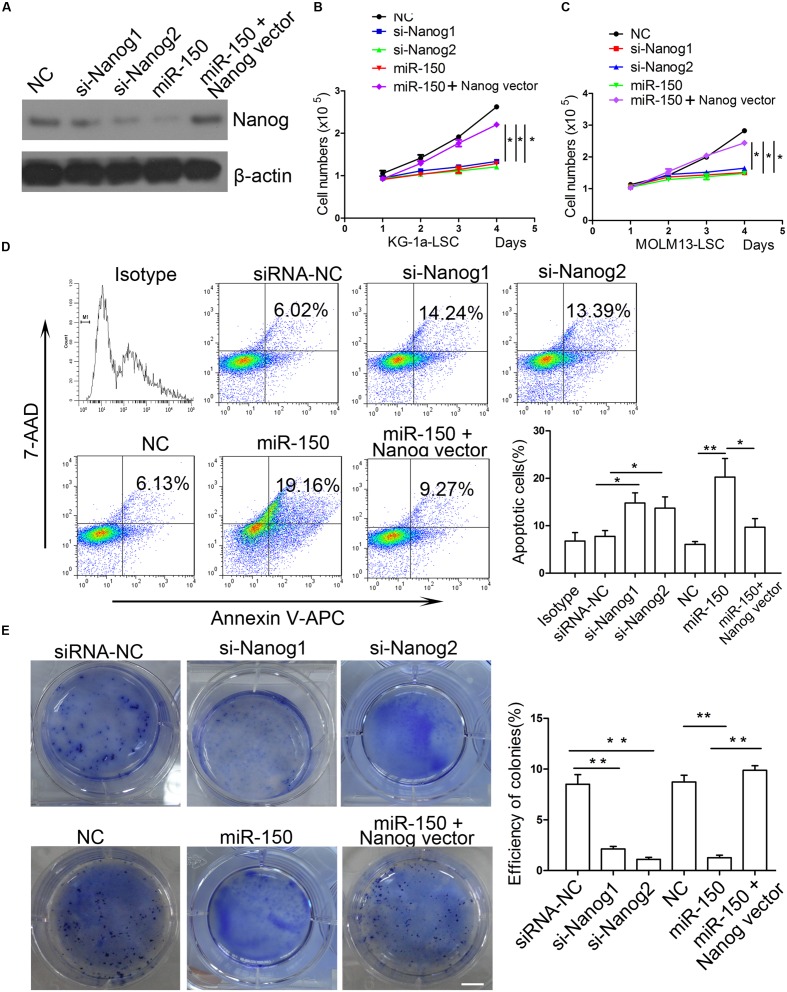
**si-Nanog recapitulates the function of miR-150 in LSCs.** **(A)** Western blot analysis of LSCs transduced with si-Nanog1 and si-Nanog2 against Nanog. β-actin was used as an internal control. **(B,C)** Proliferation analysis of LSCs transfected with siRNA against Nanog, miR-150 and Nanog vector. LSCs were transduced with siRNA, miR-150 and Nanog vector seeded into 96-well plates, and incubated for 48 h. The readings were recorded at 450 nm in a microplate absorbance reader (mean ± SD, ^∗^*P* < 0.05). **(D)** Flow cytometry apoptosis of LSCs transfected with si-Nanog1 and si-Nanog2 against Nanog, miR-150 and Nanog vector. After 48 h, LSCs were collected and the effects were evaluated by flow cytometry. NC was used as negative control. All the experiments were repeated independently three times (mean ± SD, ^∗^*P* < 0.05, ^∗∗^*P* < 0.01). **(E)** 1 × 10^3^ LSCs were transduced with si-Nanog1, siNanog2 against Nanog, miR-150 and Nanog vector. The transfected LScs were seeded into 6-well plates containing 0.3% soft agar. They were cultured for 14 days. The colonies were stained with 0.05% crystal violet for 0.5 h at 37°C and counted. All data represent the mean ± SD from three independent experiments (^∗^*P* < 0.05, ^∗∗^*P* < 0.01). Scale bars: 10 mm.

Next, we investigated whether the downregulation of Nanog inhibits LSC proliferation. To this end, both types of LSCs were infected with siRNA mimics against Nanog or siRNA-NC and Nanog was overexpressed. Western blot analysis demonstrated that the expression level of Nanog was effectively reduced by si-Nanog1 and si-Nanog2 (**Figure 5A**). miR-150 decreased Nanog expression level while Nanog vector increased its level (**Figure 5A**). To further explore whether silencing of Nanog had an impact on the proliferation of LSCs, trypan blue staining was used to evaluate LSCs proliferation. Our findings showed that the proliferation of LSCs from KG-1a and MOLM13 were suppressed after si-Nanog1 and si-Nanog2 transfecction (**Figures 5B,C**). Meanwhile, miR-150 overexpression suppressed the LSCs proliferation, which was reversed by Nanog overexpression (**Figures 5B,C**). Flow cytometry analysis demonstrated that si-Nanog1 or si-Nanog2 transfection inhibited the LSCs proliferation and promoted it apoptosis, compared with those transfected with siRNA-NC (**Figure 5D**). In addition, miR-150 overexpression increased LSCs apoptosis, which was reversed by Nanog overexpression (**Figure 5D**). Consistently, soft agar colony formation assays indicated that si-Nanog1 and si-Nanog2 significantly reduced the colonies efficiency and number of both types of LSCs (**Figure 5E**). miR-150 overexpression inhibited, but Nanog overexpression increased, the colonies number (**Figure 5E**). Collectively, silencing of Nanog using siRNA suppressed LSC proliferation and depletion of Nanog recapitulated the function of miR-150. The effect of miR-150 on LSCs proliferation was reversed by Nanog overexpression. These results confirmed that Nanog is a direct and functional target of miR-150.

### miR-150 Targets Several Stem Cell Regulatory Factors

**FIGURE 6:**
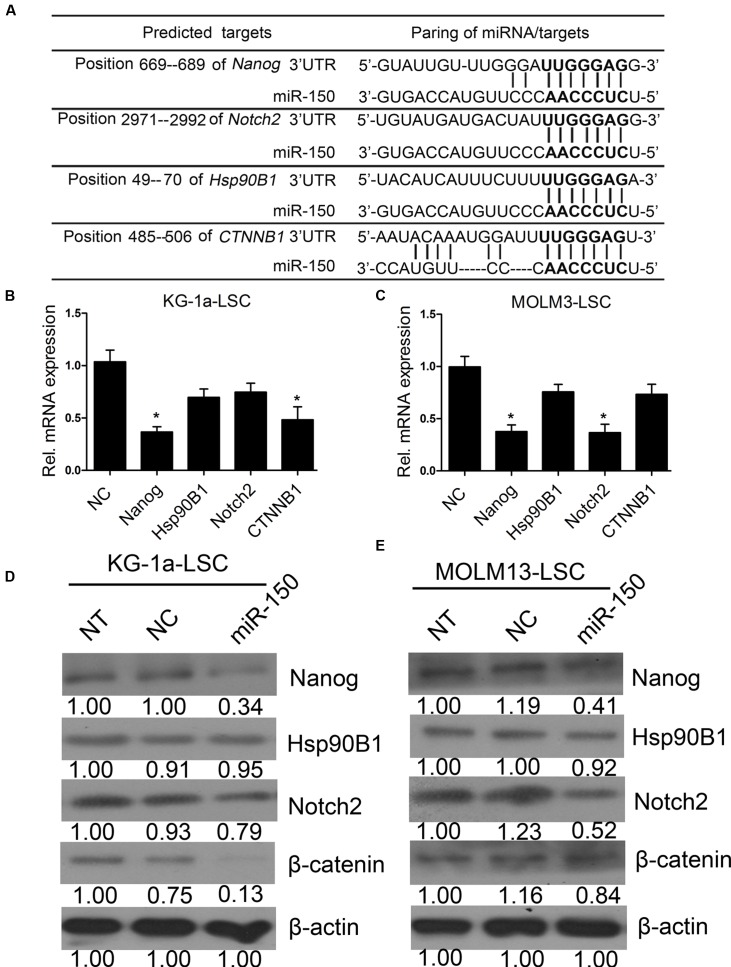
**miR-150 regulates several self-renewal genes in LSCs.** **(A)** Predicted binding targets of miR-150 to the 3′-UTRs of Nanog, Hsp90B1, CTNNB1, and Notch2. **(B,C)** qPCR analysis of miR-150’s effects on the mRNA levels of candidate genes in LSCs. β-actin was used as an internal control and the data shown are relative to the effects of NC (^∗^*P* < 0.05). **(D,E)** Western blot analysis of miR-150’s effects on the protein levels of Nanog, Hsp90B1, Notch2, and β-catenin encoded by CTNNB1 in LSCs. Densitometric values relative to NC cells are provided. β-actin was used as a loading control.

To further elucidate the molecular mechanism by which miR-150 regulates the proliferation of LSCs (**Figure 4A**), we tested the expression levels of genes including Notch2, Hsp90B1, and CTNNB1, besides Nanog (Zbinden et al., 2010; Liu et al., 2015; Zhu et al., 2015; White et al., 2016), which are known oncogenic and stem cell regulators that are implicated in leukemia initiation and progression. According to computational findings, they are targets of miR-150 (**Figure 6A**). The findings suggested that miR-150 overexpression decreased the mRNA levels of different molecules in a cell type-dependent manner. In KG-1a-LSCs, miR-150 significantly reduced Nanog and CTNNB1 levels (**Figure 6B**), whereas in MOLM13-LSCs, miR-150 additionally attenuated Nanog and Notch2 levels (**Figure 6C**). In sharp contrast, the level of Hsp90B1 remained unchanged in both types of LSCs (β-actin was used as an internal control, **Figures 6B,C**). Western blot analysis of Nanog, Notch2, β-catenin, and Hsp90B1 were consistent with the results of qPCR. As expected, Nanog levels were reduced by miR-150 overexpression in both types of LSCs, with a decrease from 50 to 80% compared with that in NC-transfected cells (*P* < 0.05, **Figures 6D,E**). The β-catenin level was sharply reduced after being transfected with miR-150, compared with transfection with NC, in KG-1a-LSCs (**Figure 6D**), while its expression level did not change in MOLM13-LSCs (**Figure 6E**). On the contrary, the Notch2 level did not change in KG-1a-LSCs after the overexpression of miR-150, but its expression level was remarkably reduced after being transfected with miR-150 compared to those transfected with NC (**Figures 6D,E**). Hsp90B1 protein levels remained unchanged in both types of LSCs transfected with or without miR-150 (**Figures 6D,E**). Taken together, these results suggest that miR-150 has a regulating effect on Nanog, Notch2, and CTNNB1 expression level, and is cell type-dependent.

## Discussion

Functional evidence demonstrates that a subpopulation of cancer cells is responsible for stem-like characteristics, such as self-renewal and limitless proliferation (Sands et al., 2013). CSCs are involved in tumor initiation, maintenance, and chemo-resistance (Ishikawa et al., 2007; Gottschling et al., 2012). Therefore, clarifying the molecular mechanism underlying the regulation of CSCs is critical for cancer disease treatment.

Over the past decade, it has been clarified that the dysregulation of miRNA expression has emerged as an essential role in leukemia, where they act as either oncogenes or tumor suppressors (Fabbri et al., 2008; Garzon and Croce, 2008). Accumulating data has demonstrated that miR-150 is associated with the development of lymphoid and myeloid lineages in human leukemia progression (He Y. et al., 2014; Stamatopoulos et al., 2015). However, the relationship between Nanog and miR-150 has not been elucidated. In this study, we identified decreased miR-150 expression levels in AML clinical samples and cell lines. Overexpression of miR-150 significantly reduced proliferation, induced apoptosis, and attenuated chemo-resistance and spheroid formation in LSCs. According to *in vivo* studies, miR-150 overexpression inhibited the tumorigenicity of LSCs. But we found that although the effect is not obvious miR-150 overexpression inhibited the tumorigenicity in LSCs. Maybe the biology role of miR-150 *in vivo* is different from that *in vitro* due to the different environment. Furthermore, miR-150 regulates the proliferation and survival of LSCs by modulating Nanog. Nanog was identified as a direct and functional target of miR-150. Consistently, the depletion of Nanog using siRNA recapitulated the observation that miR-150 targets Nanog. Therefore, our study demonstrated that miR-150 is critical for the proliferation and chemo-resistance of LSCs and that these effects were meditated by Nanog expression.

Indeed, the role of miR-150 in human cancer is context-dependent, as this microRNA functions as either an oncogene or a tumor suppressor. For example, studies have shown that the expression level is upregulated in CD19+ B cells from CLL, whereas its level was observed to be downregulated in CML and ALL (He Y. et al., 2014; Mraz et al., 2014). Furthermore, research demonstrated that miR-150 promotes proliferation and metastasis by targeting the v-src avian sarcoma (Schmidt-Ruppin A-2) viral oncogene homolog (SRC; Cao et al., 2014). In addition, *in situ* hybridisation revealed that the miR-150 expression level was decreased in breast cancer samples compared to adjacent normal cells (Huang et al., 2013). Reports by Stamatopoulos have revealed opposite prognostic significance for cellular and serum circulating miR-150 in CLL patients (Stamatopoulos et al., 2015). In our studies, we found that the miR-150 expression level was significantly decreased in CD34+ cells and LSCs compared with normal blood.

Additionally, miR-150 is warranted further investigation for other reasons. Two research groups have independently reported that the dysregulation of miR-150 expression in murine hematopoietic stem cells remarkably arrested the development of B cells at the pro-B-cell stage (Xiao et al., 2007; Zhou et al., 2007). Bruchova et al. (2007) showed that miR-150 is progressively downregulated during normal erythropoiesis. However, in 2008, another study using a novel methodology illustrated that miR-150 is moderately expressed in megakaryocyte/erythrocyte precursors and is increased as the cells undergo megakaryocytic differentiation (Lu et al., 2008). In the present study, miR-150 was shown to regulate LSC proliferation and chemo-resistance and inhibit LSC tumorigenicity.

It is of particular interest to elucidate the molecular mechanism by which miR-150 regulates hematopoietic malignancies through its target genes. The expression level of miR-150 was inversely associated with the mRNA level of MYB in MDS, which implies that MYB might be an important target of miR-150 (Xiao et al., 2007). In another study, miR-150 directly downregulated the expression of AKT2, reduced levels of phosphorylated AKT^ser473/4^, and increased levels of tumor suppressors, such as Bim and p53 (Watanabe et al., 2011). MYB, FLT3, and EGR2 have been identified as critical target genes of miR-150 in MLL-rearranged AML, while AKT2 is a direct target of miR-150 in NK/T-cell lymphoma (Fulci et al., 2007; Hussein et al., 2010; Watanabe et al., 2011; Jiang et al., 2012; He Y. et al., 2014). In our study, we found that Nanog is a direct and functional target of miR-150 in AML.

Nanog is a homeodomain protein that, along with Oct4 and Sox2, plays a role in ESC self-renewal and pluripotency (Mitsui et al., 2003; Boyer et al., 2005). In our studies, we found that Nanog is expressed in LSCs, which is consistent with Eberle’s studies (Eberle et al., 2010). In addition, studies have illustrated that Nanog2 was found in mixed lymphocytic leukemia, which suggests that Nanog2 could be involved in the regulation of leukemic stem cell functions (Eberle et al., 2010). Nanog plays a key role in CSC proliferation and clonogenic growth. For example, RNA interference-mediated silencing of NANOG leads to reduced long-term clonal and clonogenic growth and proliferation (Jeter et al., 2009). Similarly, the knockdown of Nanog was associated with a loss of proliferation, reduced self-renewal, and increased apoptosis via blocking the cell cycle progression through p53 signaling (Cao et al., 2013). In our studies, we found that Nanog is a direct and functional target of miR-150. miR-150 inhibited LSCs proliferation, which was reversed by Nanog overexpression. In addition, si-Nanog attenuated the clonogenic growth of the LSCs and promoted LSC apoptosis.

In addition to Nanog, other stemness genes including Notch2, CTNNB1 and Hsp90B1 are also important to CSCs and cancer cells. Notch2+ human pancreatic cancer Bxpc-3 and Panc-1 cells have properties of CSCs, which have a strong tumourigenic ability (Zhou et al., 2013). Depletion of CTNNB1 impaired the stem-like phenotype of renal cell carcinoma (Lin et al., 2015). Indeed, the WNT/CTNNB1 signaling is involved in regulating many types of stem cells (He K. et al., 2014). Furthermore, Hsp90B1 is expressed in various types of cancer cells including breast cancer, human osteosarcoma, CML and non-small cell lung cancer (Cawthorn et al., 2012; Li et al., 2012; Mosakhani et al., 2013; Coskunpinar et al., 2014). Accordingly, in future anti-tumor studies these stemness genes may be important targets.

## Conclusion

We have demonstrated that miR-150 is downregulated in LSCs cell lines and clinical blood samples. miR-150 overexpression can inhibit LSCs proliferation, attenuate clonal and clonogenic growth, decrease tumorigenicity, both *in vitro* and *in vivo*. Furthermore, Nanog was identified as a direct and functional target of miR-150. Silencing of Nanog recapitulated the anti-proliferation function of miR-150 and attenuated LSCs clonogenic growth. Our findings demonstrate that the miR-150/Nanog axis provides new insight into the mechanisms for eliminating LSCs and the restoration of miR-150 expression may be a potential therapeutic strategy for the treatment of AML in the future.

## Author Contributions

D-dX carried out most of the studies and performed statistical analysis, designed the study, and wrote the manuscript; YW, P-jZ, YZ, and LZ analyzed the data and carried out the bioinformatics. H-pX, C-zH, LT, B-bR, and W-yF read and revised the entire manuscript. RZ, S-hC, SW, and XW participated in the western blot design and wrote the paper. ZL, Y-hL, and X-kG conceived the study and provided professional advices. Q-yL, ZR, and J-hQ participated in the design and coordination of the study. Y-fW designed the study and revised the manuscript. All authors have read and approved the final manuscript.

## Conflict of Interest Statement

The authors declare that the research was conducted in the absence of any commercial or financial relationships that could be construed as a potential conflict of interest.

## Abbreviations

AML: acute myeloid leukemia
CLL: chronic lymphocytic leukemia
CML: chronic myeloid leukemia
LSCs: leukemia stem cells
MDS: myelodysplastic syndrome
MLL: mixed lineage leukemia
