# Supplementary material for: miR-150 Suppresses the Proliferation and Tumorigenicity of Leukemia Stem Cells by Targeting the Nanog Signaling Pathway
Source: Front Pharmacol. 2016 Nov 18;7:439. doi: 10.3389/fphar.2016.00439 (PMC5114241; doi:10.3389/fphar.2016.00439)
Supplement: Supplementary file 1 [file Table_1.doc]

**miR-150 suppresses the proliferation and tumorigenicity of leukemia stem cells by targeting the Nanog signaling pathway**

Dan-dan Xu^1,2#^, Peng-jun Zhou^1#^, Ying Wang^1,3#^, Yi Zhang^4^, Rong Zhang^5^, Li Zhang^1^, , Su-hong Chen^2^, Wu-yu Fu^3^, Bi-bo Ruan^3^, Hai-peng Xu^1^, Chao-zhi Hu^1^, Lu Tian^1^, Jin-hong Qin^1^, Sheng Wang^1^, Xiao Wang^1^, Qiu-ying Liu^1^, Zhe Ren^1^, Xue-kui Gu^6^, Yao-he Li^6^, Zhong Liu^1*^, Yi-fei Wang^1*^

Supplementary Fig.S1 microRNA analysis of miR-150 in LSCs after the LSCs were transfected with miR-150 mimic or NC.


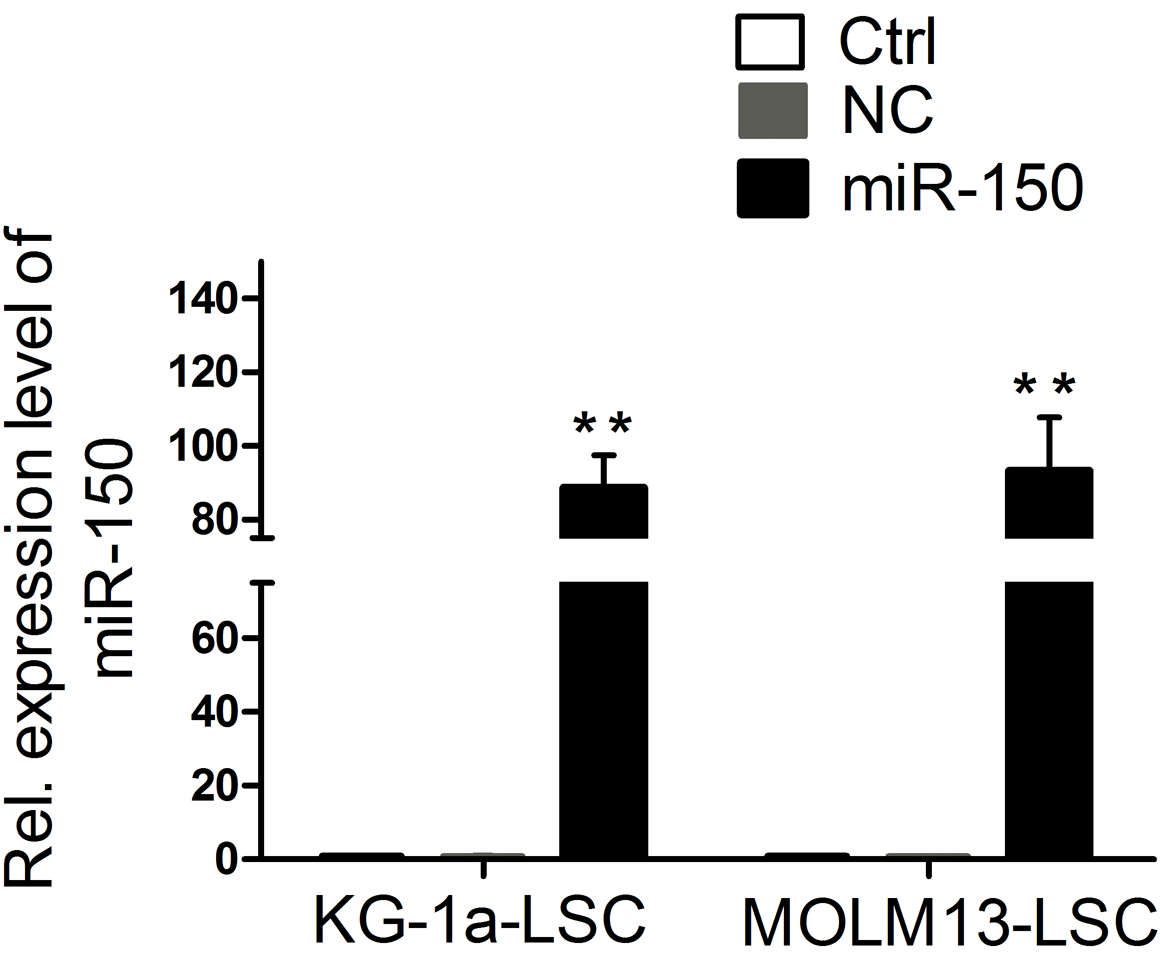


Supplementary table S1. Summary of primers.

| **Genes Primer sequences (5’-3’)** | |
| --- | --- |
| Nanog  Notch2  CTNNB1  Hsp90B1  β-actin  Nanog WT 3’-UTR  Nanog Mu 3’-UTR  NC  miR-150  si-Nanog1  si-Nanog2  siRNA-NC | F: CTGAGATGCCTCACACGGAG  R: TGTTTGCCTTTGGGACTGGT  F: TGATGATTGTGCCTTCGCCT  R: GACACAGGAGACCTGCCTTC  F: TGCGTTCTCCTCAGATGGTG  R: AGGTTATGCAAGGTCCCAGC  F: GCCAGTTTGGTGTCGGTTTC  R: GGGTAATTGTCGTTCCCCGT  F: AGGCTCTTTTCCAGCCTTCC  R: AATGCCAGGGTACATGGTGG  5′-GUAUUGUUUGGGAUUGGGAGGCUU-3′  5′-GUAUUGUUUGGGAUUCCCUGGCUU-3′  5′-UUCUCCGAACGUGUCACGU-3′  5′-GUGACCAUGUUCCCAACCCUCU-3′  5′-CCUCCAUGGAUCUGCUUAUUUU-3′  5′-CCACAAACCAUGGGAUUUAUUU-3′  5′-GCCACAAGCUGGAAUACAAUU -3′ |

Supplementary table S2. Clinical characteristic of new diagnosed AML patients

| **Characteristics (n)** | **miR-150**  **CD34+ expression, n (%) CD34- expression, n (%)** |
| --- | --- |
| Sex |  |
| Female (11) | 8 (72%) 3 (28%) |
| Male (8) | 3 (37%) 5 (63%) |
| *P* value | 0.18 |
| Age |  |
| ≤40 years old (13) | 5 (38%) 8(62%) |
| >40 years old (6) | 3 (50%) 3 (50%) |
| *P* value | 1.00 |
| FAB |  |
| M0 (4) | 2 (50%) 2 (50%) |
| M1 (3) | 1 (33%) 2 (67%) |
| M2 (8) | 4 (50%) 4 (50%) |
| M5 (4) | 3 (75%) 1 (25%) |
| *P* value | 0.25 |
| Laboratory analysis (median) |  |
| WBC (10^9^/L) | 21.3 19.5 |
| Platelet (10^9^/L) | 49 58 |
| Hemoglobin (g/dL) | 17.4 12.4 |
| BM blast, % | 41 35 |
| PB blast, % | 19 16 |
| *P* value | 0.19 |

Abbreviations: FAB, French-American-Britain subtype; WBC, white blood cell; BM, bone marrow; PB, peripheral blood. *Fisher’s exact test was used.
